# Cerebrovascular development: mechanisms and experimental approaches

**DOI:** 10.1007/s00018-021-03790-1

**Published:** 2021-03-10

**Authors:** Timothy J. A. Chico, Elisabeth C. Kugler

**Affiliations:** 1 Department of Infection, Immunity and Cardiovascular Disease, Medical School, University of Sheffield, Beech Hill Road, Sheffield, S10 2RX UK; 2 The Bateson Centre, Firth Court, University of Sheffield, Western Bank, Sheffield, S10 2TN UK; 3 Insigneo Institute for in Silico Medicine, The Pam Liversidge Building, Sheffield, S1 3JD UK

**Keywords:** Brain, Endothelial cells, Immune cells, Lymphatics, Mural cells, Vasculature

## Abstract

The cerebral vasculature plays a central role in human health and disease and possesses several unique anatomic, functional and molecular characteristics. Despite their importance, the mechanisms that determine cerebrovascular development are less well studied than other vascular territories. This is in part due to limitations of existing models and techniques for visualisation and manipulation of the cerebral vasculature. In this review we summarise the experimental approaches used to study the cerebral vessels and the mechanisms that contribute to their development.

## Introduction

The endothelial cells (ECs) of different vascular territories possess distinct molecular and function identities [1–4]. The cerebral ECs are of particular clinical relevance due to their roles in human diseases including stroke, cerebrovascular malformations and vascular dementia [5, 6]. In addition, neurological diseases including neurodegeneration and Alzheimer’s disease share risk factors with other vascular diseases and are increasingly believed to have vascular components [7, 8]. Cerebrovascular diseases together incur substantial morbidity and mortality, claiming eighteen million lives annually and consuming 8–21% of total healthcare expenditure, impacts which are increasing due to an ageing population [5, 6, 9–12]. In this review, we discuss the current understanding of cerebrovascular determination and development.

## Models and tools for studying cerebrovascular development

**Fig. 1:**
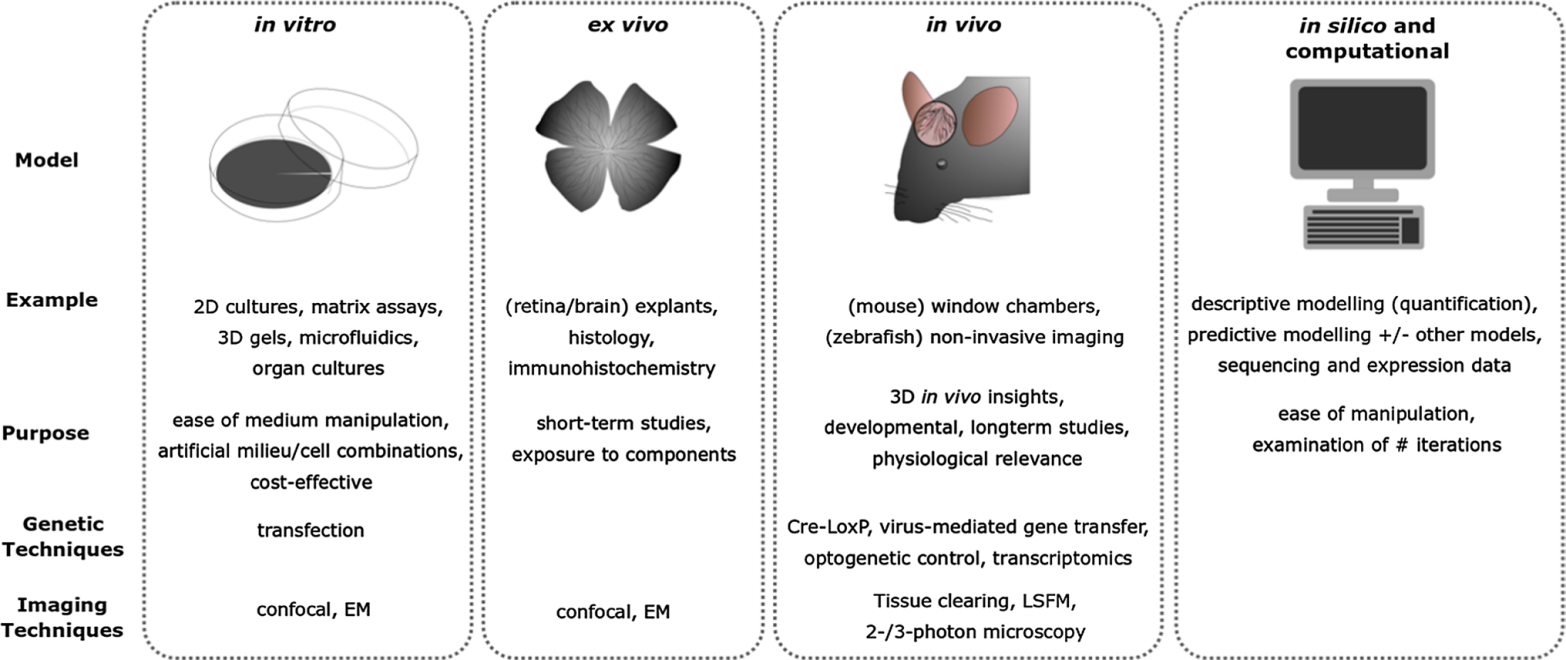
Summary of models used to study cerebrovascular development

Much of our understanding of the molecular and cellular mechanisms of EC biology comes from in vitro studies. There are arrays of such models from simple monolayers to more complex environments including matrix assays, 3D gels, and organ cultures (reviewed in [13, 14]). These allow examination of the effect of candidate molecules and mechanisms on in vitro cell behaviours; and to attempt to reproduce the in vivo milieu there have been significant technological advancements in three-dimensional and complex microenvironments. This is exemplified by microfluidic platforms mimicking the cerebral environment and cerebrovascular properties, such as neurovascular interactions [15], and the blood–brain-barrier (BBB) [16, 17]. Despite their advantages, in vitro studies provide limited insight into the influence of tissue context and the dynamic processes during development, with ECs in vitro even displaying opposite characteristics to those in vivo [18]. To understand cerebrovascular development therefore necessitates in vivo studies (Fig. 1).

Rats and mice have been extensively used for basic and translational cerebrovascular studies due to their similar neuroanatomy to human, detailed genome mapping, and sophisticated murine genetic techniques [19]. The retina provides an excellent, well characterised model to study cerebrovascular ECs. The optic vesicle forms from the CNS in early embryogenesis [20, 21] allowing study of the interactions between neurons and cerebral ECs. However, ex vivo retina studies require sacrifice of the animal, preventing in vivo imaging. In utero development and skull formation make direct observation of developmental processes challenging. This was recently addressed by a novel method mouse intravital imaging [22]. Still, imaging of the intracerebral vasculature technically challenging, requiring a surgically-created cranial window. Imaging the cerebral vessels is now possible for several weeks [23–25], with greater imaging depth achieved by 3‐photon fluorescence microscopy [26] and long wavelength reflectance confocal microscopy [27]. Despite such improvements in vivo imaging, most rodent studies of cerebral vessels remain histological. Advances such as tissue clearing [28–32] and light sheet fluorescence microscopy (LSFM) [33–35], allow whole brains to be imaged without sectioning. However, tissue processing can introduce artefacts and methods for vascular quantification, especially in 3D, are still limited. Despite this, the mouse remains the model most suitable for sophisticated genetic approaches that provide insights into the mechanisms of cerebrovascular development. These includes vascular-specific cre-recombinase [36, 37], virus-mediated gene transfer [38, 39], and optogenetic approaches [40, 41].

Zebrafish are an increasingly used non-mammalian model to study vascular formation and function. The embryos develop rapidly *ex utero*, making them accessible from the earliest stages of development [42, 43]. This allows genetic constructs to be microinjected into fertilised eggs, facilitating genetic manipulation, and transgenesis. Larval transparency and an array of transgenic reporter lines allows non-invasive observation of organogenesis in real-time. These advantages have made zebrafish an excellent model to observe in vivo cardiovascular development over time [44]. Most studies of zebrafish vascular development have focused on the trunk vasculature that has a simple and stereotypical anatomy, but the mechanisms determining the form and function of the cerebral vessels are now increasingly studied. The cerebral and trunk vasculature form as two separate vascular beds which subsequently merge [45, 46]. Despite the many advantages of zebrafish it should be noted that studying the adult zebrafish cerebral vasculature is limited by skull formation as in rodents. Thus, studying cerebral blood vessels in 3D in vivo in any adult species remains a major challenge. In addition, although the zebrafish is readily genetically manipulated to generate mutants and transgenics, the ability to induce tissue-specific gene manipulation is not as well-established as in the mouse.

In addition to in vitro, in situ, and in vivo approaches, in silico computational models have advanced our understanding on cerebrovascular development, dynamics, and disease. This is exemplified by models of cell behaviour depending on growth factors in microenvironments, which are particularly strong when linked to in vivo studies [47–49]. Similarly, large scale modelling, such as in silico studies of haemodynamic forces provide novel insights into cerebrovascular biology and disease [50–52]. To establish such predictive models, descriptive models are often required first to enable the establishment of boundary conditions. As such, (bio)medical image analysis and cerebrovascular quantification tools are not only essential to understand vascular biology, but also to allow such modelling approaches [53–56]. These models are complemented by an increasing data availability and analysis on single-cell transcriptomics, cellular diversity, cross-species diversity as well as regional specializations [4, 57–59].

Together, a wide array of tools and models exist to understand, assess, and model cerebrovascular development.

## Signalling pathways driving cerebrovascular development

The key molecular drivers of vasculogenesis, angiogenesis, vessel maturation, arteriovenous specification, and vascular remodelling are well conserved between species (reviewed extensively [60–62]). However, our understanding of these pathways is drawn from a wide range of in vitro and in vivo models, and not all have been well studied in cerebrovascular development. Below we briefly summarise these key pathways and discuss their roles in cerebral vessel development (Table 1).

**Table 1:** Overview of signalling pathways driving vascular development, their key roles and the vascular territory in which they have been shown to be functioning

Pathway	Main component(s)	Main role(s)	Cerebral vasculature	Non-cerebral vasculature	References
VEGF	*VEGF-A*	Vasculogenesis, angiogenesis	Yes	Yes	[65, 66]
	*VEGF-B*	BBB formation and vessel maintenance	Yes		[67–69]
	*VEGF-C, VEGF-D*	Lymphangiogenesis	Yes	Yes	[70, 71, 73–75]
	*VEGF-3/Flt4*	Angiogenesis	Yes		[77, 78]
	*VEGFR-1/Flt1*	“Decoy” receptor	Unknown	Yes	[79–82]
Hippo	*YAP/TAZ*	Angiogenesis, tip cell migration and barrierogenesis	Yes	Yes	[84–88]
Notch	*dll4, notch1b, notch3*	Inhibitory function in vasculogenesis, angiogenesis, arteriovenous differentiation	Yes	Yes	[89–94, 94–105]
FGF	*Fgf-2*	Mesodermal angioblasts, angiogenesis, lymphangiogenesis	Unknown	Yes	[113–117, 169]
HH	*ihh (indian)*	Yolks sac angiogenesis	Unknown	Yes	[118, 121, 122]
	*dhh (desert HH)*	BBB	Yes		[126]
	*shh (sonic HH)*	EC tubes, arteriovenous differentiation	Unknown		[123–125]
TGF-β	*Alk1, Endoglin*	EC migration, lumenization	Yes	Yes	[61, 129–135]
Wnt	*Wnt7a/b*	EC sprouting, BBB, impacts FGF signalling	Yes		[142–146, 148, 149]
Neuropilin	*Nrp1*	Arteries	Yes	Yes	[153, 154]
	*Nrp2*	Veins and lymphatics	Yes	Yes	[153, 154]
Semaphorins	Class3	Inhibition of angiogenesis	Yes	Yes	[164–167]
	Class6A	Angiogenesis	Yes		[168]
	Class7A	Angiogenesis		Yes	[171]
Netrins		EC migration and proliferation, BBB, anti-inflammatory	Yes	Yes	[174–180]
Robo/Slit	*Robo4*	Angiogenesis, cell chemotaxis, neuronal development	Yes	Yes	[183–185]
Sprouty		Inhibition of VEGF and FGF	Yes		[186, 187, 191]
PDGF		Vessel maturation by mural cell recruitment, vascular stability, neuronal activity, BBB	Yes	Yes	[192–198, 451]
Angiopoietin/Tie		Vessel maturation by mural cell recruitment, vascular stability	Yes	Yes	[199–206]
Ephrins	*Ephrin-B2*	Expressed on arteries	Yes	Yes	[216, 217, 221–223]
	*EphB4*	Expressed on veins	Yes	Yes	[218, 219, 221–223]

The Vascular Endothelial Growth Factor A-E (VEGF A-E) family of ligands are pro-angiogenic guidance cues that induce EC proliferation, migration, and vascular permeability [63, 64], mediated via VEGF receptor (VEGFR) tyrosine kinases (RTK). VEGF-A signalling is mainly mediated by VEGFR-2/Kdr/Flk1, the “master regulator” of angiogenesis required for cerebral and non-cerebral angiogenesis [65, 66].

VEGF-B acts via VEGFR-1/flt-1 and Neuropilin 1 to play a role in blood–brain barrier (BBB) formation and cerebral vessel maintenance in rodents [67, 68]. In zebrafish, VEGF-B is indispensable for neuronal and cerebrovascular development with embryonic lethality upon loss of the duplicated orthologue *vegfba*, but not *vegfbb* [69]. VEGF-C and VEGF-D mainly interact with VEGFR-3/Flt4 and promote lymphangiogenesis in vertebrates [70–72]. While VEGF-C is pivotal for embryonic lymphatic development, VEGF-D is required for inflammatory lymphatic growth and the response to brain injury [73, 74]. However, in zebrafish the roles of vegfc and vegfd are less clear-cut, with vegfd required for angiogenesis and facial lymphangiogenesis [75] and vegfc-driven VEGFR-3/Flt4 signalling playing a role in trunk angiogenesis [76].

The complex roles of receptor-based signalling are exemplified by the fact that global loss of VEGFR-3/Flt4 results in reduced cerebral EC developmental angiogenesis in mice [77], while EC specific loss results in hyper-sprouting of hindbrain and retina ECs [78]. In contrast to the above RTKs, VEGFR-1/Flt1 acts as a “decoy” receptor, binding VEGF-A with high affinity, but low RTK activity [79–82]. In zebrafish, flt1 is an important negative regulator for trunk angiogenesis [83], but whether this applies to cerebral ECs remains unclear.

Yes-associated protein (YAP) and its paralog transcriptional coactivator with PDZ-binding motif (TAZ) are Hippo pathway effectors, which mediate VEGF-VEGFR2 signalling during angiogenesis [84]. In mice, YAP/TAZ-CDC42 signalling regulates retinal vascular tip cell migration [85] and loss of YAP/TAZ leads to altered cerebral angiogenesis and vascular barrier formation [86]. Additionally, in zebrafish, YAP plays a role in cerebral and non-cerebral blood vessels maintenance [87] (reviewed in [88]).

Notch signalling counter-balances VEGF signalling, providing inhibitory functions in angiogenesis and cell proliferation [89, 90]. Notch and VEGF signalling co-ordinate *tip-stalk-cell formation* and angiogenic sprouting, with high VEGFR expression in tip cells and Notch receptor expression in stalk cells [91–95]. Tip and stalk cells of the cerebral vasculature can dynamically shuffle position in vitro and in zebrafish [49]. Alterations in Notch signalling impact cerebrovascular patterning and identity [96, 97], demonstrating Notch is essential for cerebrovascular development and maintenance.

Mathematical models have provided insights into tip-stalk-cell selection and vascular sprouting. Although some of these models compare computational findings to in vivo data, the impacts of EC microenvironments and vascular territories remain to be modelled in more depth [47, 92, 98] (for reviews see [99–101]). Notch signalling is also crucial for *arterial differentiation and maintenance* with conserved functions in different model organisms and vascular beds (cerebral and non-cerebral) [73, 82–84]. Notch dysregulation is common in human genetic cerebrovascular diseases such as Cerebral Autosomal Dominant Arteriopathy with Subcortical Infarcts and Leukoencephalopathy (CADASIL) [105–107] and Cerebral Cavernous Malformation (CCM) [108, 109] in which small cerebral capillaries develop, are remodelled, or function abnormally.

Fibroblast Growth Factor 2 (FGF-2) promotes angiogenesis in chick embryo chorioallantoic membrane [110], mouse cornea [111], zebrafish non-cerebral vessels [112, 113], and murine subcutaneous Matrigel plug [114]. Additionally, FGF-2/VEGF-C plays a role in lymphangiogenesis [115]. Whether this applies to cerebrovascular development requires further examination, which may be clinically important, since FGFR1 gain-of-function mutations are found in cerebral glioblastoma [116].

The Hedgehog (HH) morphogen family includes sonic hedgehog (shh), indian hedgehog (ihh), and desert hedgehog (dhh), which all play roles in vascular development (reviewed in [117]). ***Ihh*** is required for yolk sac blood island formation in mice [118, 119], while ***shh*** is needed for vascular tube formation in mice, birds and in vitro [120–122]. The role of ***dhh*** in angiogenesis is less clear, but *dhh* produced by cerebral ECs is required for BBB formation [123, 124]. HH impacts via angiopoietin-1 is required for cerebral EC attachment but not arteriovenous differentiation in zebrafish [125], suggesting that HH is likely to impact cerebrovascular development on multiple spatiotemporal levels.

The Transforming Growth Factor-β (TGF-β) family includes TGF-βs, bone morphogenetic proteins (BMPs), activins, and inhibins. Their role in angiogenesis is not fully elucidated as impacts are dose-dependent and TGF-β component ablation is often embryonically lethal (reviewed in [61, 126, 127]). Alk1 (type I receptor; TGFβR1; or activin receptor-like kinase 1, ACVRL1) [128, 129] and Endoglin (type III receptor; TGFβR3) [130–132] are specifically expressed in EC and are required for both cerebral and non-cerebral EC formation. Recent studies in mouse retinae [133, 134] and zebrafish non-cerebral vessels [135, 136] show that Endoglin plays a crucial role in mouse retinal angiogenesis [133] and is also implicated in hereditary haemorrhagic telangiectasia (HHT) which may cause cerebral cerebrovascular arteriovenous malformations with upregulation of VEGF signalling [133, 134]. Alk1 regulates EC migration in lumenized cerebral vessels [137], and Alk3 is required for cerebral venous identity [138].

Wnt signalling plays important roles in angiogenesis and vascular remodelling [139–142]. Wnt/β-catenin signalling is required for CNS, but not non-cerebral, angiogenesis in mice [143]. Wnt regulates FGF activity in cerebrovascular development in zebrafish [144] and Wnt7a/Wnt7b-specific signalling is required for cerebrovascular angiogenesis in both zebrafish [145] and mouse [146]. Moreover, Wnt signalling is essential for BBB formation and maintenance [147–149].

Neuropilin (Nrp) receptors are expressed in specific EC types with Nrp-1 in arteries and Nrp-2 in veins and lymphatics (reviewed in [150, 151]). Nrp-1 mediates murine cerebral EC angiogenesis and lymphangiogenesis [152, 153]. While it is believed that Nrp-1 impacts angiogenesis via interacting with VEGF, increasing evidence in mice suggests Nrp-1 might impact vascular permeability independently of VEGF [152, 154]. Conversely, in vitro studies show that VEGF can induce Nrp-1 [155]. Understanding the impact of Nrps is complicated by the finding that the Nrp-1 cytoplasmic domain is dispensable for cerebral and non-cerebral EC angiogenesis, but promotes arteriovenous separation [156]. Nrp1 and Nrp2 double-knockouts in mice are embryonically lethal, and lack both cerebral and non-cerebral vasculature [157].

In zebrafish, all four Neuropilins play roles in angiogenesis, with loss of nrp1b and nrp2a leading to cerebral vascular abnormalities [158, 159]. A recent study suggests Nrp-1 signalling is indispensable for vascular development and that Nrp-signals via VEGF [69]. Increasing evidence also suggests Nrp influence cerebral angiogenesis via TGFβ and PDGF [127, 160].

Semaphorins were originally identified as axon-guidance cues but also mediate angiogenesis. ***Class 3 semaphorins*** inhibit angiogenesis in both non-cerebral [161–163] and cerebral ECs in mice [164]. While ***Semaphorin 6A*** regulates angiogenesis in the mouse retina [165], ***Semaphorin 7A*** mediates angiogenesis in mouse non-cerebral ECs [166]. Semaphorins also impact angiogenesis of non-cerebral ECs in the zebrafish [167, 168], but cerebral vessels have not been studied.

Netrins are axonal guidance and attraction cues [163, 163] and play a role in angiogenesis, influencing EC migration and proliferation in CAM, zebrafish trunk vessel, and mouse retinae [171–173] as well as anti-inflammatory functions [174–176. In the cerebral vasculature, netrins play a role in BBB integrity [170, 177, 178].

The Roundabout receptors (Robo) and Slit ligands impact angiogenesis, EC chemotaxis, and neuronal development (reviewed in [179]). Robo4 is essential for angiogenesis in vivo in the zebrafish trunk vasculature and mouse ex vivo retina [180, 181]. Cerebrovascular roles of Robo/Slit are yet to be defined.

Sprouty ligands inhibit Ras–ERK MAPK signalling, creating a negative feedback loop for VEGF and FGF signalling [182, 183] that inhibits angiogenesis. Studies in zebrafish, mice, and human show different Sprouty ligands are expressed in the brain [184–186] (reviewed in [187]), but their roles in cerebrovascular formation are undefined.

Platelet-derived growth factor (PDGF) is a chemoattractant mitogen. PDGF-B and its receptor PDGFRβ [188–190] are required for recruitment of pericytes to cerebral vessels [191] and play a role in blood–brain-barrier (BBB) development, post-stroke, and neurodegenerative diseases [192–194].

Angiopoietin ligands and their RTKs Tie receptors play crucial roles in mural cell recruitment and vascular stability [195–197]. Angiopoietin-1 promotes vascular stability, while Angiopoietin-2 decreases stability and promotes vascular remodelling [198–202]. An underlying mechanism for this difference in function is differential Tie-2 receptor phosphorylation [203]. Human EC and mouse data suggests that Tie-1 regulates Tie-2 intracellular trafficking [204]. Angiopoietin-1 regulates cerebrovascular permeability in human brain microvascular endothelial cells in vitro [205], while studies in fish showed that Angiopoietin-1 impacts brain size [206]. Angiopoietin-2 is dysregulated in mouse cerebral cavernous malformation (CCM) [207], but its inhibition rescues arteriovenous malformation (AVM) in a hereditary haemorrhagic telangiectasia (HHT) mouse model [208]. Additionally, macrophage-derived Angiopoietin-Like Protein 2 aggravates post-ischemia inflammation [209] and Angiopoietin like-4 mediates capillary cerebral amyloid angiopathy, a feature of some human neurological diseases [210].

The ephrin-B2 ligand and EphB4 receptor are crucial regulators of vascular morphogenesis (reviewed in [211]). Ephrin-B2 is expressed on arteries and EphB4 on veins. Loss of function results in defective angiogenesis in cerebral vessels in mice [212, 213]. EphB4 modulates VEGFR-2 signalling through phospho‐ERK1/2 in cerebral ECs [214, 215]. Ephrin-B2 is pro-angiogenic in mouse cerebral ECs [216], and Ephrins also play a role in neural patterning (reviewed in [217–219]) as well as neuropathologies [220, 221].

## The role of blood flow in cerebrovascular development

Blood flow impacts EC gene expression and cell behaviour via flow-derived forces [222, 223]. Studies in mice show that hemodynamic forces directly impact cerebrovascular topology by remodelling cerebrovascular (cornea [224] and retinal [225]) vessels. Hemodynamic forces appear to impact EC migration rather than cell death in all vascular beds, and Notch signalling regulates this in zebrafish [226] and mice [227–229]. Blood flow also impacts EC gene expression and epigenetics, of relevance to cerebrovascular diseases such as aneurysm, stroke, and neurodegeneration [5, 7, 230, 231] but also crucial for stabilization of arterio-venous identity after molecular initiation [213, 232] (reviewed in [233–235]). In zebrafish, although arterio-venous differentiation of trunk vessels is understood in great detail, cerebrovascular identity establishment is less well described [226, 236–239].

Blood flow also plays a crucial role in *vascular lumenization*. How this is achieved in different vascular beds and in different species is not fully understood. In zebrafish, different mechanisms of non-cerebral vessel lumenization are proposed, including ‘*vacuole fusion*’ [240, 241] and ‘*inverse membrane blebbing*’ [242] in ISVs, ‘*lumen ensheathment*’ in common cardinal veins (CCVs) [243], and ‘*hollowing*’ of the dorsal cord [244]. How cerebral vessels are lumenized is unclear and only indirect evidence suggests cerebrovascular lumenization by ‘*budding*’ [245]. In mice and human, lumenization is even less well understood and studies suggest ‘*intracellular vacuoles’* and ‘*cord-hollowing*’ are potential mechanisms [246–248]. Lastly, blood flow plays a role in brain ventricular expansion [249], indirectly impacting cerebral vessel development via brain morphogenesis.

## The role of cilia in angiogenesis

*Immotile* primary cilia on EC sense blood flow and are disrupted by shear stress in human ECs [250] as well as on non-cerebral ECs in zebrafish [251] and mice [252, 253]. Loss of primary cilia in mice impairs EC vascular integrity and homeostasis [254] and heart development leading to aneurysms and atherosclerosis [255], while endothelial primary cilia are dispensable for vascular development but are atheroprotective [253]. In zebrafish the subcellular localization of cerebrovascular primary EC cilia is independent of blood flow and cardiac contractions [256]. Primary cilia also impact EC polarization and migratory behaviour in the mouse retina [257] and regulate hematopoietic stem and progenitor cell specification in the zebrafish aorta-gonad mesonephros [258].

Ependymal motile cilia are required for brain development and CSF flow directionality in zebrafish [259]. Similarly in mice, cilia are essential for the brain ependyma and choroid plexus development [260, 261], and adult CSF flow [262].

Loss of cilia in *embryonic* zebrafish results in cerebrovascular EC defects [256, 257, 263], cerebral haemorrhage [125, 264], and reduced arterial vSMCs of non-cerebral vessels [265]. It remains unclear whether cilia loss impacts cerebrovascular vSMC and the BBB. Interestingly, loss of cilia in *juvenile and adult* zebrafish does not result in gross vascular defects in cerebral and non-cerebral vascular beds [266].

## Non-endothelial cells required for cerebrovascular development

Pericytes and vascular smooth muscle cells (vSMCs) collectively known as vascular mural cells (MCs), ensheath vessels abluminally, providing vascular stability and homeostasis [197, 267, 268]. Previously it was believed that trunk MCs are mesoderm derived, while cerebral MCs are neural crest and mesoderm derived [97, 269]. Recent studies suggests transdifferentiation of macrophages into pericytes, vSMCs, and ECs may contribute to vascular MCs [270–272]. Such transdifferentiation was recently shown in mice [273] but whether this occurs in other species is unknown. With technical advancements in mouse transgenesis and in vivo imaging, it is possible to study cerebral MCs in developing [274] and adult [275] mice, showing that MCs are heterogeneous and structurally plastic. In both zebrafish and mice, impairment of mural cell coverage leads to BBB breakdown [192, 276, 277].

Radial glia, also called astroglia, are neural stem cells, support neuronal migration, and regulate CNS angiogenesis [278–280]. Radial glia, moreover, impact astrocyte differentiation, thus contribute to neurovascular unit functionality [281]. As radial glia in zebrafish harbour high regenerative capacity, zebrafish are a suitable model to study CNS regeneration [282–284].

Astrocytes are specialised cerebral and spinal cord glial cells that ensheath blood vessel with astrocytic end-feet. They provide structural support and as part of the BBB, play a role in tripartite synapse homeostasis and regulate blood flow [285–292]. Astrocytes play a key role in neuron maintenance as well as ionic and osmotic brain homeostasis [293–295]. Increasing evidence suggests astrocytes are a potential link for vascular contributions to cognitive impairment and dementia [294]. A murine study found that neurons, which are ensheathed by astrocytes, form a migration scaffold for blood vessels with astrocyte-derived VEGF being crucial for angiogenesis [296], while another showed that oxygen provided by retinal ECs promotes astrocyte differentiation [297].

The role of immune cells in EC development, inflammation, repair, and cancer has gained increased attention. Macrophages are of particular clinical interest as tumour associated macrophages (TAMs) are associated with cancer prognosis [298, 299] and TAMs contribute to brain malignancies [300–302] and glioma progression [303]. In intracerebral haemorrhage (ICH), macrophages play a role in the secondary post-stroke phase and represent a therapeutic target [304, 305]. While mice have been the main model of choice to examine cerebral haemorrhage, zebrafish models also allow study of ICH [306–308]. Macrophages also monitor neuronal activity and impact neuronal structural remodelling in mice [309] and zebrafish [310], and thus may impact cerebral ECs indirectly via neurons. Macrophages can act as cellular chaperones guiding cerebral vessel formation [311] and clear apoptotically pruned cerebral ECs [312] during zebrafish development. During tissue repair, zebrafish macrophages mediate vascular repair in cerebral ECs [313] and have been shown to migrate faster when in contact with trunk vessels compared with non-vascular cells [314], though whether this applies to cerebral ECs is unknown.

Many studies on *neutrophils* examine their roles in cancer and the tumour microenvironment (review [315, 316]). Neutrophils play a role in VEGF signalling and matrix-metalloproteinase secretion, contributing to tumour angiogenesis, and metastasis in mice [317, 318], while inhibition of VEGF signalling decreased tumour angiogenesis but increased neutrophil-mediated metastasis in zebrafish [319]. Very few publications examine the role of neutrophils in cerebrovascular development, and neutrophils do not enter the brain under normal conditions [320].

Neurons and ECs use common guidance molecules for repulsion and retraction (*e.g. ephrins, semaphorins, slits and netrins,* see above), and share functional and molecular similarities in pathfinding, growth, migration, and differentiation [321–323]. This structural parallel growth of vessels and neurons, called *neurovascular congruency* [324], is important in shaping mouse cerebral vessels [322, 325–327], but it has been suggested that vessels and neurons are independently patterned in more complex 3D tissues such as mouse whiskers [328]. Additionally, studies in murine non-cerebral ECs showed that motor-neurons impact vascular patterning in the spinal cord [329], while blood vessels are important regulators of neural stem cell properties [330]. Zebrafish studies of cerebral *neurovascular congruency* suggest the vasculature is required for hindbrain development [331] and ganglia projections [332]. Conversely ablation of oxytocin in the hypothalamo-neurohypophyseal system results in defects of vascular patterning [333] and motor neurons were shown to be essential for vascular pathfinding in non-cerebral vessels [334]. Besides these structural and molecular interactions, cerebrovascular ECs and neurons interact functionally via *neurovascular coupling* (NVC; or functional hyperemia). NVC increases local cerebral blood flow in response to increased neuronal activity, orchestrated by ECs, neurons, astrocytes, and vSMCs [335–337]. Evidence in human and rodents showed that NVC is impaired with age [338–341] and neurodegenerative diseases such as Alzheimer’s [8, 342–345].

How NVC develops in humans is currently unclear due to the difficulty in age-matching of patients and the lack of long-term studies [346, 347]. Studies in rats showed absent or negative blood-oxygen-level-dependent (BOLD) signals in functional magnetic resonance imaging (fMRI) with increased BOLD signals 2–3 weeks postnatally [348, 349]. In zebrafish NVC develops between 6-to-8dpf and high glucose exposure impairs NVC, mirroring human diabetes [350, 351].

## The role of the extracellular matrix in cerebrovascular development

The vascular basal lamina (BL) is a thin, dense cross-linked network of extracellular matrix (ECM) proteins forming a thin barrier (mainly *laminin, collagen IV, nidogen/entactin, and heparin sulfate proteoglycans (HSPGs)*). It is synthesised by ECs, astrocytes, and pericytes (reviewed in [352–354]). Previously the BL was considered a passive scaffold providing structural support for ECs, astrocytes, and pericytes by adhering to the BL via integrins and proteoglycans. New interest emerged due to its role in angiogenesis, BBB integrity, and pathology (reviewed in [355, 356]) and the finding that BL-composition is highly tissue-specific and altered in disease [357–360]. ***Laminins*** are pivotal for basement membrane integrity in the eye [361], retinal angiogenesis [362], and BBB integrity [363, 364]. A murine study suggested the role of BL laminins in retinal angiogenesis is mediated via recruitment of microglia [365]. *Laminin/integrin-induced Dll4/Notch-signalling* is crucial in tip cell formation in retinal cerebrovascular ECs in mice [366] and in vitro in primary human ECs [367]. **Collagen IV** is essential for organism-wide BL maintenance but dispensable for initial assembly [368]. Loss of Col4a1 leads to cerebral-specific haemorrhage and porencephaly [369, 370]. In zebrafish, loss of collagen IV impacts axonogenesis [371], but its impact on ECs is unknown. ***Nidogen/entactin*** links the laminin and collagen IV networks [372]. Loss of nidogen-1 results in neurological defects and structural alteration of brain capillaries with BL thinning in mice [373], while loss of nidogen-2 resulted in no obvious phenotypic effect [374]. Murine nidogen-1 and nidogen-2 co-regulate and are enriched in ECs [375], but this did not examine cerebral ECs. Cardiac and pulmonary haemorrhage upon nidogen loss is reported, but again no data on cerebrovascular ECs is available [376]. In zebrafish four nidogen family members (*nid1a*, *nid1b*, *nid2a* and *nid2b*) are predicted to exist, but only loss of *nid1a* has been studied with a phenotype of reduced body length [377]. Three different ***HSPGs*** are found in the vascular BL, namely *perlecan, agrin, and collagen XVII* [378]*. Perlecan* modulates cell adhesion, proliferation, and growth factor signalling [379]. Perlecan is lost within hours after cerebral ischemic stroke in non-human primates [380]. In mice, perlecan is not required for early angiogenesis, but loss of perlecan results in cerebrovascular defects, brain hemorrhage [381], and profound cardiovascular defects [382, 383]. This suggests perlecan plays a role in angiogenesis and/or vascular maintenance, especially in cerebral vessels. In contrast, the C terminus of perlecan, named "endorepellin", inhibits angiogenesis [384]. Morpholino studies in zebrafish suggested loss of perlecan results in defects of somitogenesis and angiogenesis, exemplified by loss of circulation in cerebral and trunk vessels. Although the authors showed that perlecan (heparan sulfate proteoglycan 2/ hspg2) impacts EC proliferation and migration in trunk vessels, it is unclear whether this is the case for cerebrovascular ECs [385, 386]. ***Agrin*** plays a role in the development of the body posterior, CNS [387], and retina in zebrafish [388], but the role in EC and cerebrovascular EC-specific is unclear. In mice, agrin promotes heart regeneration [389] and contributes to cerebrovascular EC adherens junctions and thus EC barrier properties [390], and agrin is required for BBB formation in chicken and rat [391]. Other ECM components, such as ***metalloproteases, metalloproteinases, and ADAMS***, play a role in angiogenic sprouting/remodelling as well as the angiogenic switch during carcinogenesis (see reviews [392–394]). Their cerebrovascular-specific roles are far from understood although MMP-2 and MMP-9 are upregulated after cerebral ischemia in non-human primates [395, 396].

## The blood brain barrier, choroid plexus and cerebral lymphatic development

The brain undergoes major structural developmental remodelling, by invagination in mammals and eversion in zebrafish [397]. Higher vertebrates (birds, amphibians, and mammals) have four brain ventricles, while fish have two [398, 399]. Each ventricle has a choroid plexus [398], harbouring ependymal cells, which are the major source of cerebrospinal fluid (CSF) [400, 401], with CSF potentially contributed by liquid flux through capillary walls [402, 403].

The CSF serves a protective role and provides the CNS with nutrients and waste removal. Factors that contribute to CSF movement are respiration [404, 405] and head movement [406] in humans. Murine studies show cilia play a role in ependymal flow [407, 408], while studies in zebrafish show contraction, body movement, and cilia all play a role in CSF flow [259].

The *blood–brain-barrier (BBB)* protects the brain from pathogens, neurotoxic molecules, and lipophilic elements [293, 409]. The BBB is formed by inter-EC junctions and specialized transporters of cerebrovascular ECs, to provide a barrier between blood and interstitial fluid [1]. Recent transcriptional profiling studies in mice examined cell types and zonation of the cerebral vasculature [4] and how the BBB is impacted in models of CNS disease (stroke, multiple sclerosis, traumatic brain injury and seizure) [410]. In all the examined CNS diseases cerebral ECs shift towards non-cerebral EC expression patterns. These studies also examined non-cerebral tissues, allowing examination of cerebrovascular-specific EC properties. The ability to study BBB formation in vivo and over long periods of time made zebrafish a favourable model for barrierogenesis [411–415], with recent work showing that barrierogenesis and cerebrovascular angiogenesis occur in parallel [416].

Following the discovery of meningeal lymphatics in mice [417, 418], similar findings in zebrafish [419–421], non-human primates, and humans followed [422]. In zebrafish the meningeal lymphatics regulate meningeal angiogenesis [419]. Similarly, the brain parenchyma was thought to be widely devoid of parenchymal lymphatics [423], but this was challenged by the finding that lymphatics invade brain parenchyma in response to injury to guide vascular regeneration [424]. The concepts of cerebral waste clearance were additionally challenged by the finding of the “*glymphatic system*” in mice in 2012 [425, 426]. This clearance system is made of perivascular channels formed by astroglial cells and is mainly active during sleep and reduces with age (reviews [427–430]).

## Concluding remarks and future perspectives

With the availability of different models our knowledge of cerebral vascular development has increased substantially. Although the general mechanisms and molecular regulation of ECs development and function are now understood in great detail [60–62, 431–433], the degree to which these are conserved between models and between the cerebral and other vascular territories is far less complete. More sophisticated experimental models such as organoids, 3D cell co-culturing systems, 3D printing, as well as in silico computational models [434–437] will aid our mechanistic understanding of the diversity of cerebrovascular ECs.

It is increasingly clear that individual mechanisms and cellular components interact with each other. The field would benefit from understanding these interactions in different vascular beds, and how these change in development and disease.

Complementary to these new models, further improvements of imaging techniques and image analysis approaches allow for cutting edge data acquisition, and performance of meaningful objective quantifications which can handle large datasets. Medical imaging techniques are being applied to pre-clinical studies, such as Optical projection tomography (OPT) [438–440], Magnetic Resonance Imaging (MRI) [441, 442], Optical Coherence Tomography and Angiography (OCTA) and Nuclear Magnetic Resonance (NMR) [443], or combining cutting edge techniques such as LSFM and OPT [444]. Importantly, imaging techniques are evolving from descriptive to quantitative [55, 445] and integrating information from other tissue [446–449]. This quantification will also impact future drug screening and pharmacodynamics studies, helping to identify strategies to treat human cerebrovascular diseases.
